# A transcriptome-based association study of  growth, wood quality, and oleoresin traits in a *slash pine*  breeding population

**DOI:** 10.1371/journal.pgen.1010017

**Published:** 2022-02-02

**Authors:** Xianyin Ding, Shu Diao, Qifu Luan, Harry X. Wu, Yini Zhang, Jingmin Jiang

**Affiliations:** 1 Research Institute of Subtropical Forestry, Chinese Academy of Forestry, Hangzhou, China; 2 National Forestry and Grassland Engineering Technology Research Center of Exotic Pine Cultivation, Hangzhou, China; 3 Umeå Plant Science Centre, Department of Forest Genetics and Plant Physiology, Swedish University of Agricultural Sciences, Umeå, Sweden; 4 CSIRO National Collection Research Australia, Black Mountain Laboratory, Canberra, Australia; The University of North Carolina at Chapel Hill, UNITED STATES

## Abstract

Slash pine (*Pinus elliottii* Engelm.) is an important timber and resin species in the United States, China, Brazil and other countries. Understanding the genetic basis of these traits will accelerate its breeding progress. We carried out a genome-wide association study (GWAS), transcriptome-wide association study (TWAS) and weighted gene co-expression network analysis (WGCNA) for growth, wood quality, and oleoresin traits using 240 unrelated individuals from a Chinese slash pine breeding population. We developed high quality 53,229 single nucleotide polymorphisms (SNPs). Our analysis reveals three main results: (1) the Chinese breeding population can be divided into three genetic groups with a mean inbreeding coefficient of 0.137; (2) 32 SNPs significantly were associated with growth and oleoresin traits, accounting for the phenotypic variance ranging from 12.3% to 21.8% and from 10.6% to 16.7%, respectively; and (3) six genes encoding PeTLP, PeAP2/ERF, PePUP9, PeSLP, PeHSP, and PeOCT1 proteins were identified and validated by quantitative real time polymerase chain reaction for their association with growth and oleoresin traits. These results could be useful for tree breeding and functional studies in advanced slash pine breeding program.

## Author summary

Slash pine is an important source of original timber and resin production on commercial forest plantations. It is necessary to implement precise breeding strategies to improve timber quality and resin yield. However, little is known about the species’ molecular genetic basis. Using a transcriptome dataset with sequencing from 240 individuals in the slash pine population, we combined multiple approaches (based on gene variation, expression variation and co-expression network) to dissect the genetic structure for slash pine major breeding traits. We found that the research population could be divided into three genetic groups with a mean heterozygosity of 0.2246. We also found that six genes with important functions in slash pine resin synthesis and timber formation through association studies. Four new SNPs associatation with the average ring width were also discovered. Our results provide new insights into the molecular genetic basis of important traits in slash pine and provide a comprehensive method for association analyses of conifer tree species with large genome.

## Introduction

The natural range of slash pine (*Pinus elliottii* Engelm. var. *elliottii*) is the most restricted of all major southern pines; it only extends from southern South Carolina to central Florida and westward to southeastern Louisiana. Slash pine has been introduced to many countries for timber production and oleoresin tapping. The species has been extensively introduced into China, Brazil, Chile, Argentina, Venezuela, South Africa, New Zealand, and Australia [1]. Slash pine planting is nearing 3 million ha in China, where breeding programs began more than 40 years ago and there are large breeding populations consisting of several hundred selections. The growth and wood properties have been improved to some extent through two-cycles of breeding activities [2,3], however, selection intensity is low and gene recombination for the first two-cycles of breeding activities is limited [4]. For advanced genetic improvement of slash pine, marker-assisted breeding (MAB), genomic selection (GS) and gene editing would accelerate the breeding progress. To implement MAB, increase the effectiveness of GS and explore the gene editing, detection of candidate genes for quantitative traits would be preferable through association study [5]. A transcriptome-referenced association study (TRAS, also called transcriptome wide association study (TWAS)), with a genome-wide association study (GWAS-like) approach was recently carried out as part of the improvement program for slash pine breeding populations in China [6].

Association studies are becoming the experimental approach of choice to dissect complex traits in many organisms from model plant systems to humans [4]. The candidate gene-based association approach has several important advantages for complex trait dissection in many coniferous forest tree species, because they are outcrossing with less population structured, adequate levels of nucleotide diversity, rapid decay of linkage disequilibrium. For clonally propagated speces, more precise evaluation of phenotype are possible.

Recent advances in genome sequencing and bioinformatics technology have enabled the development and efficient assay of large numbers of single nucleotide polymorphisms (SNP) markers, even in the absence of reference genome sequences [7,8]. Genetic variations in many complex traits alter protein abundance by regulating gene expression, and many studies have highlighted gene expression patterns could be a key tool for linking DNA sequence variation to phenotypes [9–11]. Relationships between gene expression and trait performance can be investigated through association transcriptomics which could reduce the sequence need by the entire genome [12]. The transcriptome obtained by single molecule long-read sequencing was recently used as a reference sequence for scoring population variation in both sequence SNPs (SNP markers) and expression level variants (gene expression markers, GEMs); this method was termed a TRAS and holds promise as a GWAS-like approach in trees [6–8]. GWAS has been used in discovering the genetic basis of complex traits in many different plants, including *Arabidopsis thaliana* [13], *Oryza sativa* [14], and *Populus trichocarpa* [15], conifers [16,17]. The detection of associations between gene expression variation and trait variation may be particularly useful in complex traits in slash pine and may compensate for the deficiency of GWAS [18,19].

To further refine the results of association studies, many co-expression network approaches have been successfully used for the identification of hub genes related to mutations [20]. Even when the phenotypic effect of each SNP is very small, grouping important genes according to their expression trends indicates that many of these genes may participate in the same physiological process or regulatory network [21].

Controlling false positives or negatives in GWAS has accelerated the development of methods for integrating multiple types of data sources. Combining GWAS with differential gene expression data has improved our understanding of the genomic structure of complex traits and the genetic basis for trait variation at the gene expression level. For *Picea glauca*, a combined association analysis and co-expressed network approach was used to dissect the genetic architecture of wood properties [20]. Integrative analysis of transcriptome and GWAS data was proposed as a strategy to identify hub genes associated with milk yield trait in buffalo [22], and it provided new insights for identifying the drought response mechanisms of *Pinus massoniana* needles and roots [23]. The aims of the present study were: (1) to study the genetic architecture of growth, wood, and resin production traits in a Chinese slash pine breeding population using a panel of transcripts with diverse functions and expression profiles; and (2) to integrate association studies with co-expression network analysis to shed light on the hub and key genes that were strongly associated with traits.

## Methods

### Plant material

**Fig 1:**
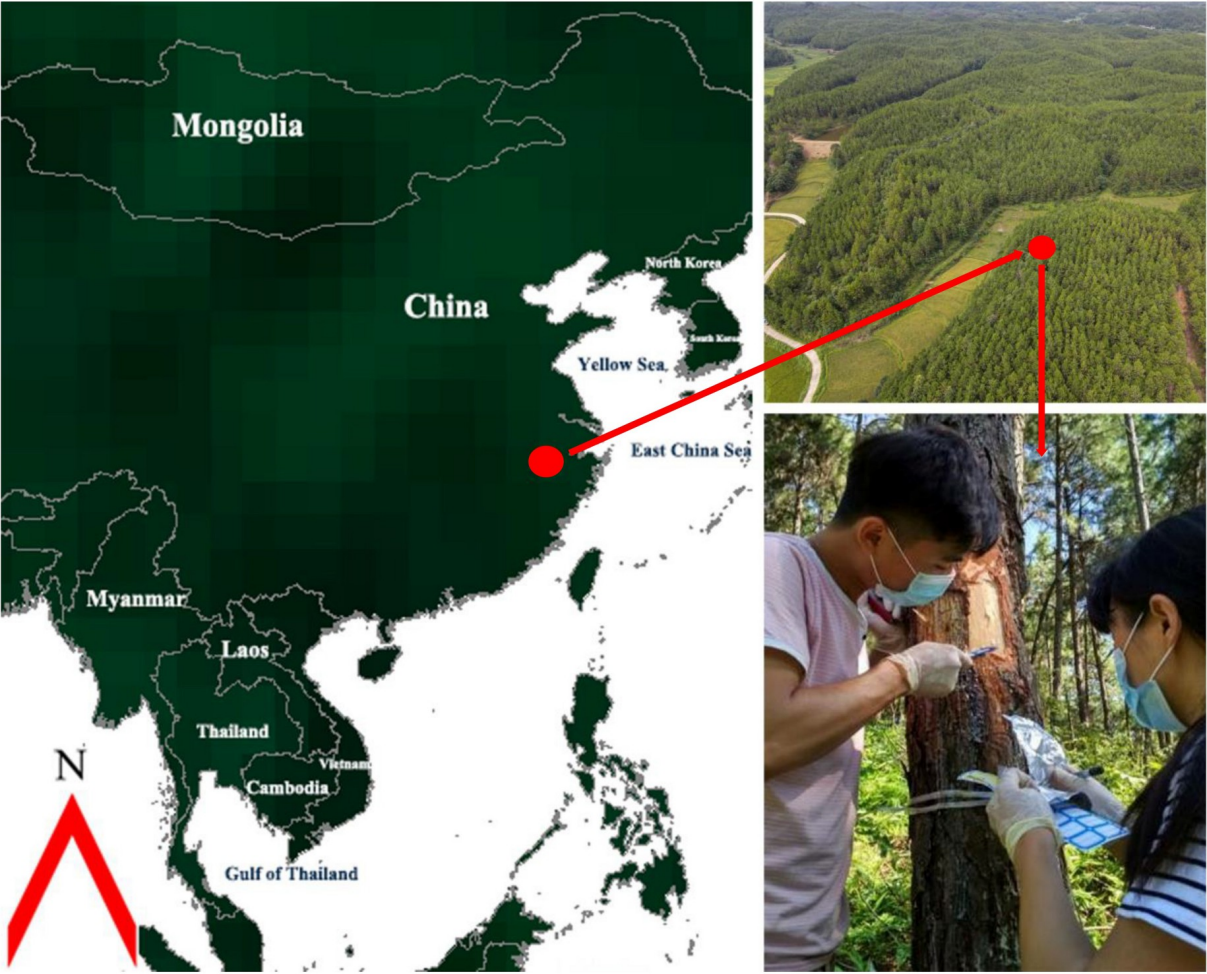
Geographic location of the RNA collection plantation and the photos showing collection of developing secondary xylem tissues of slash pines in this experiment. (Left: satellite map obtained from website https://www.naturalearthdata.com/http://www.naturalearthdata.com/download/110m/cultural/110m_cultural.zip; Up right: Overview of the plantation; Below right: the first and second authors; Photoed by Qifu Luan).

We selected one individual each from the 240 open-pollinated families, planted at the Changle Forest Farm in Zhejiang Province, China (30°27’N, 119°49’E) (Fig 1). The trial was established in March 1994 using a randomized complete block design with six blocks, with each block containing plot of six individuals per family. Trees with their diameter at breast height (DBH) of the selected tree close to the mean DBH of the family were selected. The mother trees of those families were from several germplasm collections planted in southern China, which were originally sourced from the natural range of the species in North America through international cooperation before 1990.

In July of 2019, 0.5–1 mm of deep, freshly developing secondary xylem tissues adjoining the cambium layer were harvested after removing the bark and phloem. Samples were immediately placed in liquid nitrogen after harvesting and then stored at -80°C for subsequent RNA extraction.

### RNA extraction and quality control

Total RNA from each sample was isolated using the RNAprep Pure Plant Kit (TIANGEN Biotech Co., Ltd., Beijing, China). Total RNA concentration and integrity was detected using an UV/visible spectrophotometer and an Agilent 2100 Bioanalyzer (Agilent Technologies, Santa Clara, CA, USA). Only qualifying RNA samples (A260/A280 > 2; RNA integrity number > 8; 28S/18S > 1; RNA concentration > 150 ng/μL, total RNA mass > 2.5 ng) were used for sequencing and constructing cDNA libraries.

### Next-generation sequencing and quality control

All the RNA of the xylem from 240 adult slash pine individuals was used for poly(A)+ selection using oligo (dT) magnetic beads (610–02, Invitrogen, Carlsbad, CA, USA). The selected RNA was eluted in water and RNA-seq libraries were constructed using the ScriptSeq Kit (Illumina, San Diego, CA, USA). Library quality was assessed using the Qubit 2.0 Fluorometer (Life Technologies, Carlsbad, CA, USA) and the Agilent 2100 Bioanalyzer. The effective concentration of the RNA-seq libraries were quantified using quantitative polymerase chain reaction (Q-PCR). The librariy with an effective concentration >2 nM were sequenced using the Illumina NovaSeq 6000 platform by Biomarker Tech (Beijing, China). The raw reads were filtered to remove low quality reads and adapters. Reads with “N” base contents exceeding 10% were also filtered out. Finally, reads with a quality score of Q<10 that accounted for >50% of the entire length were removed.

### SNP calling and genotyping

Clean reads were then mapped to the full-length transcript sequences developed in our former work [6] using the RNA-seq software STAR [24], and GATK [25] was adopted for SNP calling. Identified SNP sites were further annotated and analysed for their effect on the expression level of genes and associated protein products. SNPs satisfying following criteria were retained: (1) ≤3 consecutive single base mismatches within the range of 35 bp and (2) the quality value of SNPs after sequence depth standardization was >2.0. According to the number of alleles per SNP site (i.e., the number of different bases supported by sequencing reads), SNP sites were divided into homozygous SNP sites (only one allele) and heterozygous SNP sites (two or more alleles).

### Quality control and annotated SNPs

Several filtering steps were performed to further improve the quality of called SNPs: (1) only biallelic SNPs kept, (2) SNPs with a call rate (“missingness”) <95% or with a minor allele frequency (MAF) <0.05 removed, and (4) individuals with a call rate <50% were also removed. After these filtering steps, a total of 53,229 high-quality SNPs remained for further analysis. We used snpEff software v4.5 [26] to annotate these SNPs. We used Beagle v5.0 [27] to impute the missing genotypes.

### Genetic diversity and population structure analyses

PLINK software v1.9 [28] was used to estimate the observed heterozygosity (H_o_) and expected heterozygosity (H_e_) and to perform principal component analysis (PCA). The results were visualized by R software v4.0.2. We used ADMIXTURE v1.3.0 [29] to determine the genetic structure of the slash pine population. The preset ancestral population numbers ranged from K = 1 to K = 10. The K value corresponding to the minimum value of the cross-validation error rate was used as the optimal clustering. The unrooted phylogenetic tree was drawn using ITOL (https://itol.embl.de/upload.cgi). Pairwise genetic variation parameters (e.g., *F*_*st*_ index estimates and *Nei’*s unbiased genetic distance) among subpopulations/genetic clusters were estimated using VCFtools v0.0.13 [30]. The SNPs-based kinship matrix was estimated by the software Genome-wide Complex Trait Analysis (GCTA; v1.92.1) [31].

### Phenotyping

In total, we measured six traits (S1 Table), including one oleoresin yield trait (OY/g), three growth traits (DBH/cm; tree height, Ht/m; average of cumulative ring width, ARW/cm), and two wood quality traits (wood density, WD/g·m^-3^; average amplitude, AM/%). The resin was collected from the sunny side of the tree trunk. A special plastic pipe with an aperture of 18 mm was fixed at the drilling hole in the trunk to collect oleoresin, then the OY in the pipe was measured [3].

The DBH and Ht traits for all individuals in the trial were measured. ARW was indirectly measured by a resistograph instrument, which is a nondestructive and rapid instrument for detecting wood density according to the measured AM curve (strongly correlated to WD), and ARW were also calculated by the AM curve [32,33]. In addition, we obtained wood core samples and then used the saturated water content method [34] to estimate the WD.

### SNPs association for phenotypic traits

TASSEL (v5.0) [35] was used to identify associations between SNPs and phenotypic traits with the mixed linear model (MLM) approach incorporating the kinship matrix and population structure. *P* value thresholds of 9.4e-07 (0.05/53,229) and 1.9e-06 (0.1/53,229) were used to determine the SNP significance. Deviations of *P* values from expectation were evaluated using quantile-quantile (*QQ*) plots.

### GEMs association for phenotypic traits

The relationship between gene expression and the six traits were examined in a linear regression model with the TPM (transcripts per million, normalized gene expression units) values for each unigene as the dependent variable and phenotypic traits as independent variables, and *R*^*2*^ and significance (*P*) values were calculated for each unigene model using R v4.0.2. The results were visualized by the CMplot package in R software, which highlight unigenes with logarithmic transformation *P* values ≥ 2.

### Co-expression analysis

Co-expression networks were constructed using the weighted gene co-expression network analysis (WGCNA; v1.7.3) package in R [36]. The transcriptome data were standardized and then the MAD (median absolute deviation) of each gene was calculated, and the genes with MAD values in the top 5% were selected as gene groups with large inter-sample variation for WGCNA construction. Ultimately, the TPM values of 6193 unigenes were retained out of 259,396 unigenes from 240 slash pine individuals for co-expression network analysis. The first step was to construct a matrix of pairwise correlations between the 6193 pairs of unigenes across 240 samples. We then raised these correlations to a soft-thresholding power (*β* = 8) to approximate the scale-free topology within the network. From these scaled correlations, we constructed a topological overlap matrix (TOM) between all unigenes, which summarizes the degree of shared connections between any two unigenes. To identify modules of coexpressed unigenes, topological overlap-based dissimilarity was constructed [37] and then used as the input for average linkage hierarchical clustering. A value of 0.2 was selected to cut the branches of the dendrogram using the dynamic tree-cutting algorithm, resulting in a network containing 17 modules. Each module was represented by a color and contained 54 to 2229 unigenes. Each module is summarized using the “module eigengene” (ME), which is the first principal component of gene expression, and is the most representative expression pattern within the group of genes. We then associated the eigengene for each module with the phenotypic traits. The networks were visualized, and the degree value was calculated using Cytoscape v3.8.2.

### Gene ontology (GO) annotation and enrichment analysis

Since there is no GO annotation file for slash pine yet, we annotated the genome from scratch (S2 Table). First, all unigenes were mapped to the SwissProt, nonredundant (NR), Pfam, and eggNOG databases using local BLAST v2.5.0 and DIAMOND v4.1.0. We then transformed the protein ID to the GO ID with a PYTHON v3.8.5 script. Finally, we obtained the corresponding GO terms from http://geneontology.org/docs/download-ontologyhttp://geneontology.org/docs/download-ontology/%23subsets and used the clusterProfiler package of R software to perform GO enrichment analysis [38]. GO enrichment was derived with Fisher’s exact tests and a *q* value cut-off of < 0.05.

### qRT-PCR analysis

According to phenotypic measurement, 10 samples with the highest and the lowest values of each trait were selected for quantitative analysis. Primer pairs were designed for six genes, *AP2/ERF*, *TLP*, *PUP9*, *SLP*, *HSP*, and *OCT1*. The cDNA was amplified according to the Takara rr820a kit (Takara, Shiga, Japan), and the expression levels of the genes were calculated using the 2^-ΔΔCt^ method [39]. The *UBI* [40] gene was used to normalize the transcript profiles.

## Results

### Global RNA-seq and genetic variation analysis of slash pine xylem tissues

**Fig 2:**
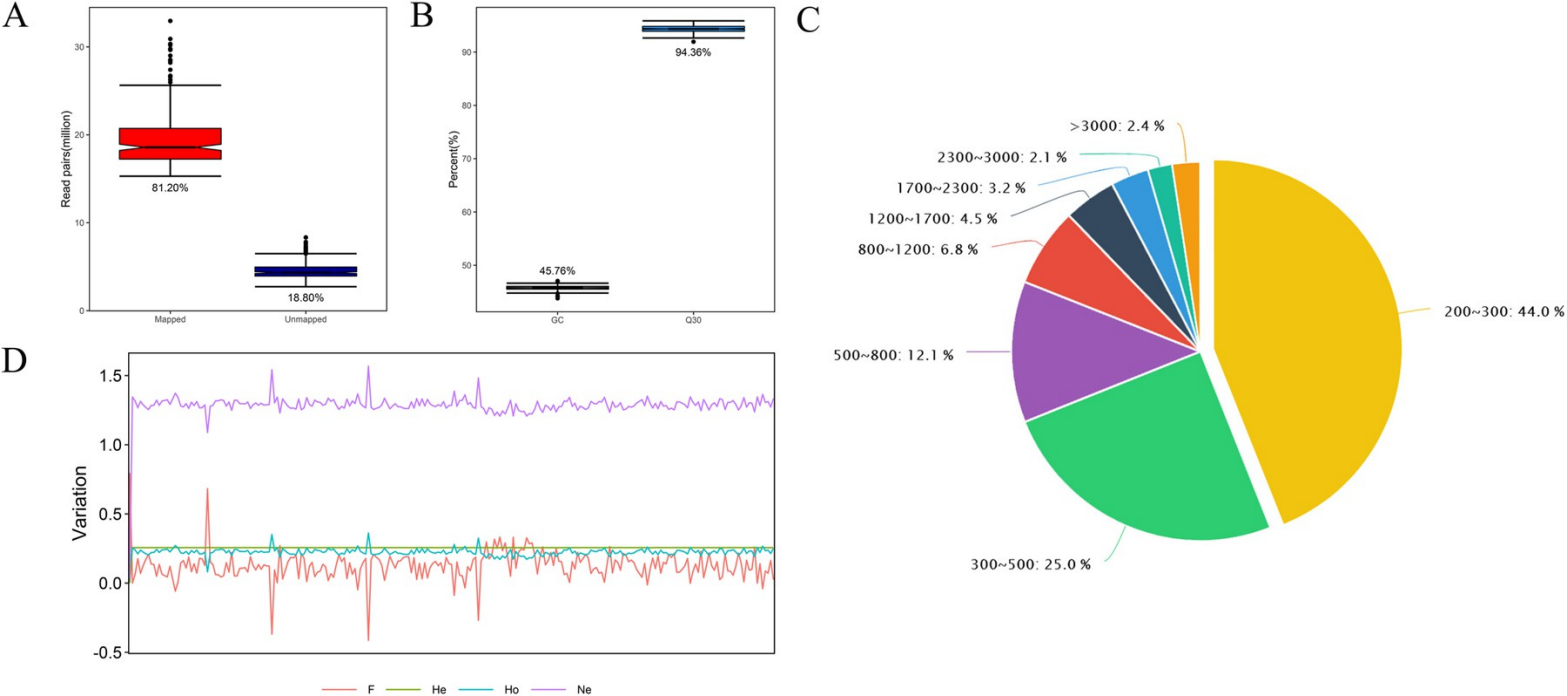
Transcriptome analysis of 240 selected slash pine trees. **A** Percentages of read pairs aligned to the reference sequence. **B** GC content and Q30 proportion. **C** The proportion of unigenes with different sequence lengths. **D** Transcript variation in the slash pine population (*F*_*is*_, *H*_*e*_, *H*_*o*_, *N*_*e*_). The vertical axis shows the proportion of each variation value relative to all values.

On average, 19.6 million (81.20%) read pairs per sample were successfully mapped to the reference sequences, suggesting high completeness of the reference transcripts (Fig 2A and S3 Table). The average GC content and Q30 value for the clean reads were 45.76% and 94.36%, respectively (Fig 2B). A total of 259,396 nonredundant unigenes consisting of sequences of various lengths were assembled, and the majority (92.4%) were sequences from 200 to 1700 bp (Fig 2C).

A mean of 2.4 million paired-end reads were sequenced per individual in 240 samples, resulting in 974,733 SNPs, with a mean SNP density of 2.87 SNPs per unigene. A core of 53,229 SNPs with a call rate > 95% and a MAF ≥ 0.05 were retained. The core SNPs, including 35,026 (65.80%) nonsynonymous SNPs and 18,203 (34.20%) synonymous SNPs, were subsequently used for association analysis and genetic diversity analysis (S4 Table).

In Fig 2D, the mean *H*_*o*_ value was lower than the *H*_*e*_ value; the *H*_*o*_ value ranged from 0.0815 to 0.3622, while *H*_*e*_ ranged from 0.2556 to 0.2584. The values of the effective number of alleles (*N*_*e*_) ranged from 1.0888 to 1.5679 with a mean of 1.2911. The mean of inbreeding coefficient (*F*_*is*_) value was estimated as 0.1373.

### Population structure and kinship of slash pine breeding population

**Fig 3:**
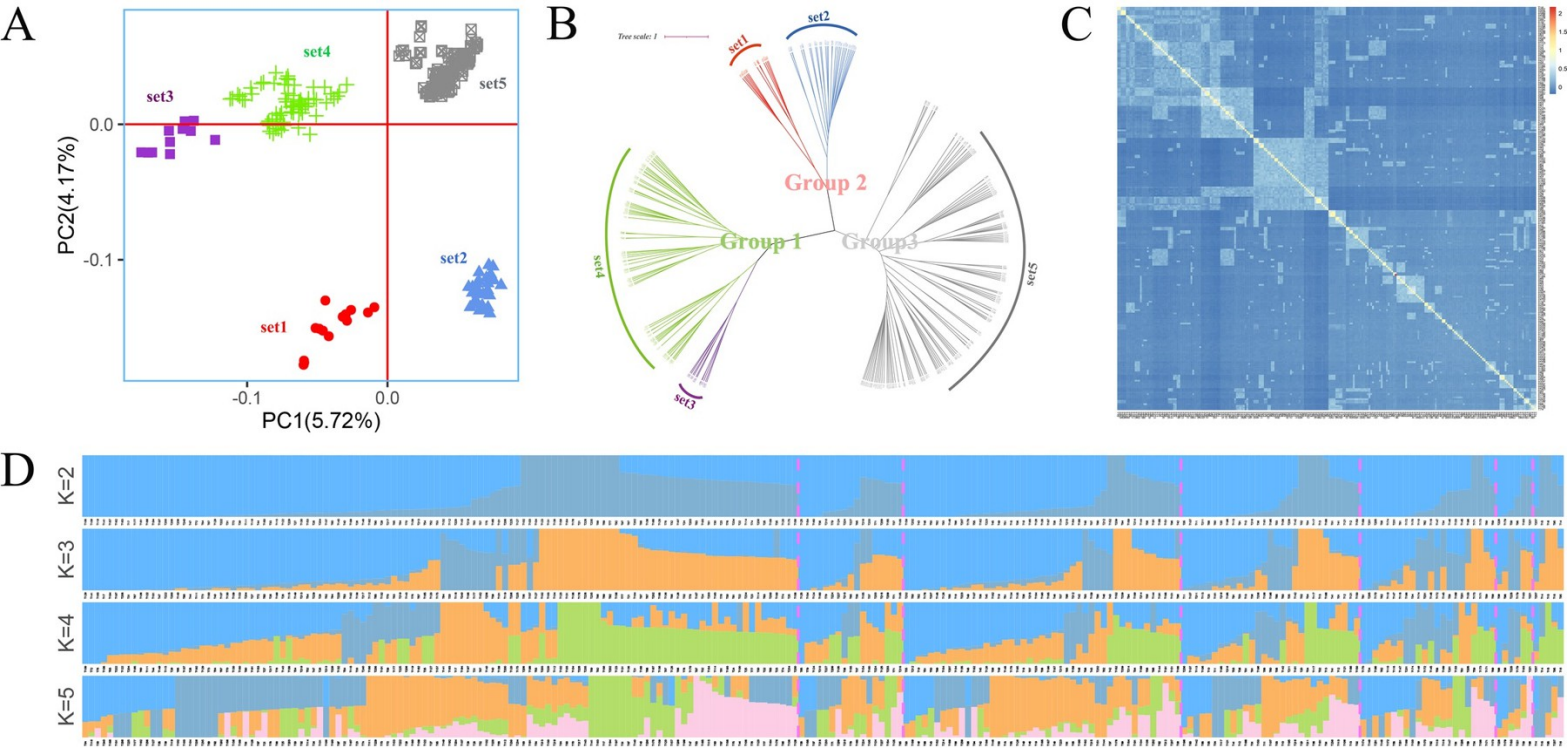
Genetic structure analysis of *P*. *elliottii* Engelm. **A** PCA scatter plot. All genotypes were grouped into five sets. **B** Phylogenetic tree analysis of the 240 slash pines based on 53,229 SNPs. All individuals were divided into three groups, which is consistent with the PCA results. C Kinship relationship among 240 slash pine individuals. Each small square represents kinship between two samples. The kinship values are colore-coded with large values approching red. D Population structure distribution of 240 slash pines at K values from 2 to 5, respectively.

PCA clearly revealed five genetic clusters (sets) (Fig 3A). Set1 and Set3 only contain 14 and 11 individuals respectively (S5 Table). We further examined the phylogeny with a distance matrix that was obtained by calculating the genetic distance between individuals (Fig 3B). It is observed that all branches lead to three genetic groups.

ADMIXTURE software [29] was used to estimate the ancestry proportions for each individual. Apparently, K = 3, corresponded to the lowest cross entropy (Figs 3D and S1), indicating that the clustering results were consistent with the phylogenetic tree (S6 Table). This indicates our slash pine breeding population may be collected from three genetic clusters in the United States. Furthermore, we estimated the genetic diversity within and between three groups. The *F*_*is*_ of group1 was 0.1335 while those of the other two groups were -0.0285 and 0.0453 (Table 1). The genetic differentiation index *F*_*st*_ and *Nei’*s unbiased genetic distance between the groups showed low values from 0.060 to 0.172 (S7 Table).

**Table 1:** Genetic diversity parameters at the group level inferred by ADMIXTURE.

Group	π	I	*H*_*e*_	*N*_*e*_	*F*_*is*_
Group 1	2.28×10^−2^	1.4566	0.2423	1.2746	0.1335
Group 2	2.32×10^−2^	1.4274	0.2481	1.3324	-0.0285
Group 3	2.29×10^−2^	1.5154	0.2476	1.3113	0.0453

*π*, nucleotide diversity; *F*_*is*_, inbreeding coefficient; *H*_*o*_, observed heterozygosity; *H*_*e*_, expected heterozygosity; *I*, Shannon’s diversity index; *N*_*e*_, effective number of alleles.

The kinship matrix among the 240 individuals was presented as a heatmap (Fig 3C) with the genetic relationship values between individuals were mainly from -0.2 to 0.25.

### Genome-wide association analysis

We identified 32 significant SNP-trait associations with growth (Ht, DBH and ARW) and OY traits. These SNPs were distributed among 31 unigenes (Table 2) and explained the phenotypic variance of 12.3% to 21.8% and 10.6% to 16.7% for growth and OY trait, respectively. OY had the most 17 SNP associations while none of SNPs were significantly associated with wood properties in this study (Table 2). Notably, there were no functional annotation results for the four SNPs that were significantly associated with ARW, suggesting that they may be genes unique to the slash pine genome.

**Table 2:** SNPs with a significant effect on phenotypic variance in slash pine.

Trait	SNP position^a^	Allele	*P* value	R^2^	Effect^b^	Annotated function
OY	sc305561.graph_c0_seq1_1774	A/C	3.75E-09	0.167	NS	Prolyl endopeptidase-like
	sc23805.graph_c0_seq1_66	A/T	4.79E-08	0.141	ST	-
	sc85433. graph_c0_seq1_133	C/T	6.58E-08	0.158	NS	-
	sc151754. graph_c0_seq1_120	A/T	1.22E-07	0.132	SN	-
	sc99405. graph_c0_seq1_99	A/G	1.67E-07	0.129	SN	-
	sc142395.graph_c0_seq1_178	T/C	2.40E-07	0.145	NS	TLP
	sc174584.graph_c0_seq1 _790	C/G	3.39E-07	0.157	NS	Uncharacterized protein
	sc311225.graph_c0_seq1_529	C/T	3.43E-07	0.141	NS	AP2/ERF
	sc319377.graph_c0_seq1_ 1371	G/A	4.28E-07	0.120	NS	Lycopene beta cyclase
	sc366749.graph_c0_seq1_94	C/A	5.12E-07	0.137	SN	ZIP metal ion transporter family
	sc222929. graph_c0_seq1_363	C/T	5.29E-07	0.118	SN	-
	sc124698. graph_c0_seq1_94	G/A	5.35E-07	0.137	NS	-
	sc119969. graph_c0_seq1_120	T/G	1.07E-06	0.111	NS	-
	sc213033. graph_c0_seq1_219	C/T	1.09E-06	0.111	SN	-
	sc326758.graph_c0_seq1_289	A/G	1.19E-06	0.143	NS	Tubulin-folding cofactor D
	sc179223.graph_c0_seq1_31	T/C	1.77E-06	0.125	NS	-
	sc118731.graph_c0_seq1_45	T/C	1.84E-06	0.106	SN	-
Ht	sc165335.graph_c0_seq1_422	T/G	3.04E-07	0.156	SL	SLP
	sc155531.graph_c3_seq1_269	T/A	3.91E-07	0.154	NS	-
	sc48339.graph_c0_seq1_37	C/G	7.86E-07	0.146	NS	HSP-like protein
	sc148216. graph_c2_seq1_164	A/G	9.59E-07	0.130	NS	-
	sc319077.graph_c0_seq1_ 1238	A/G	1.10E-06	0.143	NS	JCGZ_14506
	sc356450.graph_c0_seq1_1778	A/G	1.47E-06	0.140	NS	SELMODRAFT_447985
	sc334091.graph_c0_seq1_1827	T/A	1.54E-06	0.139	NS	OCT 1
DBH	sc338111.graph_c0_seq1_130	T/C	2.24E-07	0.145	NS	Mitochondrial pyruvate carrier 1
	sc336796.graph_c0_seq1_427	C/A	8.34E-07	0.147	NS	ELP
	sc332943.graph_c0_seq1_514	T/C	9.41E-07	0.146	NS	PUP 9
	sc332943.graph_c0_seq1_348	T/C	1.49E-06	0.141	SN	PUP 9
ARW	sc124302.graph_c0_seq1_170	A/G	2.29E-11	0.218	NS	-
	sc203621.graph_c0_seq1_94	A/G	2.75E-09	0.169	NS	-
	sc87798.graph_c0_seq1_110	T/G	6.62E-08	0.137	NS	-
	sc253463.graph_c0_seq1_107	T/C	2.98E-07	0.123	NS	-

^a^The SNP position, where the “s” stands for SNP, the number represents the SNP position on the corresponding unigene, *R*^*2*^ phenotypic variation explain rate, Allele (the first sequence is the mutated sequence, and the second is the reference sequence)

^b^The Effect refers annotation of the SNP, where NS stands for Nonsynonymous coding, SL stands for Stop-loss, SN stands for Synonymous coding, and ST stands for Stop-gain.

### Annotations of significant SNPs associated with OY, Ht and DBH

**Fig 4:**
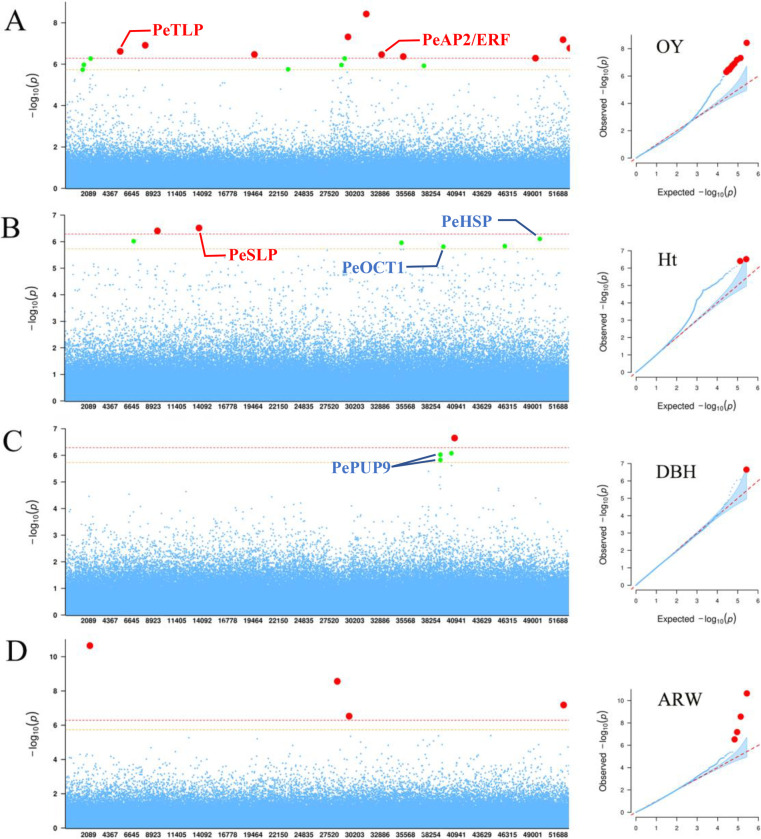
SNP-based association analysis. The x-coordinate is the transcriptome length. **A** The results of the associations between SNPs and oleoresin yield. **B** The results of the associations between SNPs and Ht. **C** Associations between SNPs and DBH. **D** Associations between SNPs and ARW. The right is the QQ plot, and the left is the Manhattan plot. Significant (*P* < 1.9e-06) and extremely significant (*P* < 9.4e-07) threshold lines are represented by orange and red dashed lines, respectively. Significant and extremely significant SNP sites are represented by enlarged green and red solid points, respectively.

Among 17 SNPs identified to associate with OY (Fig 4A and Table 2), a SNP mutated from APETALA2(AP2)/ethylene-responsive transcription factor (ERF) was identified on gene c311225.graph_c0. This is similar to *P*. *taeda* [41] and *P*. *massoniana* [8] that AP2 transcription factor (TF) was also found to associate with OY. We also identified a significant SNP on gene c142395.graph_c0 that was annotated as thaumatin-like protein (TLP), which belongs to the PR-5 family of defensive proteins that are found in higher plants and play an important roles in the plant defense system and response to pathogens [42].

Seven significant SNPs associated with Ht were detected (Fig 4B and Table 2). The most significant SNP (*R*^*2*^ *=* 0.156, *P =* 3.04E-07) was located on gene c165335.graph_c0 and was annotated as subtilisin-like protein (SLP). The SLP family is a large group of serine proteases [43] and is the key participants in the eukaryotic peptide signaling maturation pathway [44,45]. One heat shock-like protein (HSP) was significantly associated with Ht in this study. The HSP family is present in almost all living species and carries out important biological functions including the regulation of cell division, chaperone activity, signaling, and transcriptional and translational control [46]. The organic cation/carnitine transporter 1 (OCT 1) that belongs to the major facilitator superfamily was also associated with Ht.

We identified four significant SNPs from three genes associated with DBH, (Fig 4C and Table 2). Cytokinins (CKs) are a major group of phytohormones that regulate plant growth, development, and stress responses. Purine permease (PUP)-mediated uptake of adenine can be inhibited by CK, indicating that it is a transport substrate [47].

### Gene expression marker associations analysis

**Fig 5:**
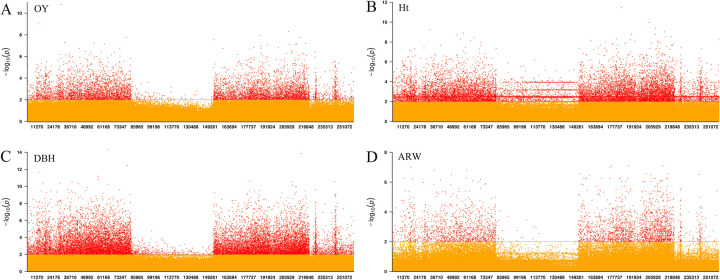
GEM-based association analysis, as a Manhattan plot. **A** Associations between SNPs and oleoresin yield. **B** The results of the association between SNPs and tree height. **C** Associations between SNPs and diameter at breast height. **D** Associations between SNPs and ARW. Significant and extremely significant threshold lines are represented by orange and red dashed lines, respectively. Significant and extremely significant SNP sites are represented by enlarged green and red solid points, respectively.

We performed a TWAS to correlate GEMs with trait variation (Fig 5). There were 24,183, 17,912, 16,546 and 7,189 unigenes that significantly (*P* < 0.01) associated with Ht, DBH, ARW, and OY respectively (S1 File). DBH showed the strongest association with gene expression levels, followed by ARW, Ht and OY, as illustrated by the distribution of significant associations for genes in the transcriptome. We performed GO enrichment analysis to identify GO categories that were significantly enriched with significant GEMs (S2 File). Genes significantly related to OY were mainly enriched in methyltransferase activity, histone acetyltransferase activity, fatty acid metabolic process, and tricarboxylic acid cycle. The corresponding unigenes were annotated as tocopherol cyclase, oxidoreductase family, methyltransferase, and sterile alpha motif domain.

The genes significantly related to Ht mainly were enriched in cellular amino acid metabolic processes and cellulose microfibril organization. The unigenes were mainly annotated as AUX/IAA family, F-box domain, cytochrome P450, cupin domain, cellulose synthase, and pectinesterase. Notably, eight TFs were identified from six families, including CBF-B/NF-YA, Trihelix ASIL2, bHLH35, bHLH30, and GTE10. For DBH, genes related to oxidoreductase activity, transferase activity, GTP binding and hydrolase activity were enriched. Twenty-two relative unigenes were annotated as TFs in 12 families such as bHLH, ERF, MADS-box, and HSP. Corresponding gene functions such as cytochrome P450, CK, F-box domain, TLP, no apical meristem (NAM) protein, cyclin and inhibitor of apoptosis-promoting Bax1 were also associated with DBH. For ARW, the main GO enrichment is acid-amino acid ligase activity, catechol O-methyltransferase activity and peroxisome. The unigenes were annotated as NAM, cell division control protein, AUX/IAA family, cytochrome p450, HSP, F-box protein and G-box-binding factor. In addition, 13 TF families containing 22 TFs were identified (S1 and S2 Files).

### Construction of gene co-expression networks

**Fig 6:**
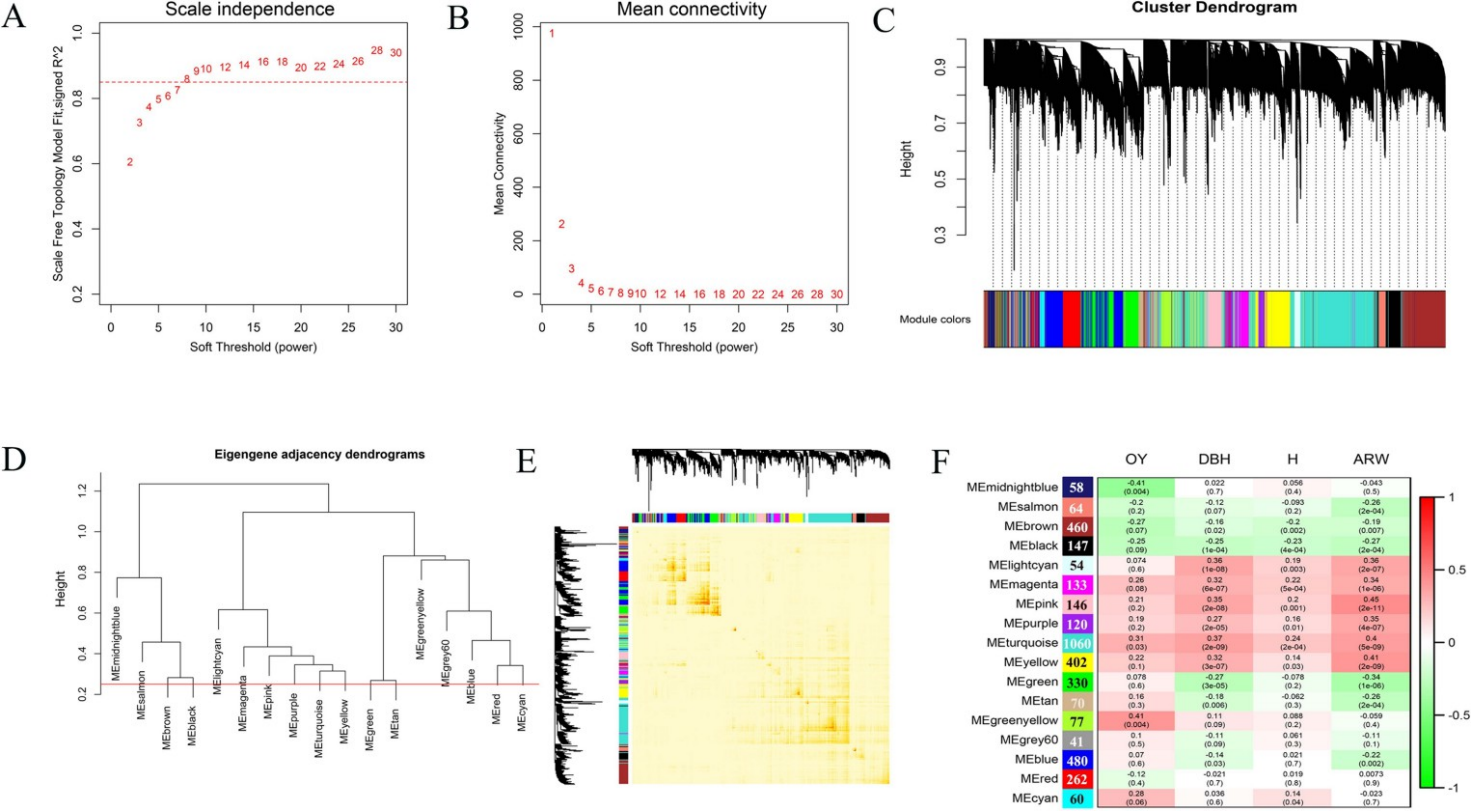
Construction of gene co-expression networks with WGCNA. **A B** Soft threshold (β value) determination that makes the network close to scale-free; the red dotted line is drawn at 0.85. **C** The genes were clustered, and then the tree was clipped into different modules using the dynamic shearing method (the minimum number of genes in the module was set to 30). **D** Eigengene cluster tree. The module below the red line (at 0.2), which indicates correlation >0.8, was merged in the next step. **E** TOM map. The rows and columns represent individual genes, and deep yellow and red represent high topological overlap. **F** Correlation heat map between the modules and four traits. The values in the box represent the correlations and *P* values. The numbers of genes in the modules are shown as the different colored boxes.

To identify the transcript regulation modules of the four growth and OY traits, a WGCNA was constructed based on the RNA-seq data of 240 slash pine accessions (Fig 6). For the 6,193 screened transcripts, a total of 17 co-expression modules were detected, excluding the grey module (containing 2,229 transcripts), which contained all genes that did not clearly belong to any other module (Fig 6C). The minimum module size was set to 30, and the eigengenes between all of the modules had an extremely low correlation (the height threshold was set to 0.25) (Fig 6D). The size of modules ranged from 41 (grey60 module) to 1060 transcripts (turquoise module) (Fig 6F). The heatmap shows the TOM of all genes in the analysis (Fig 6E). Module-trait relationships of WGCNA showed that OY was positively correlated with the module MEgreenyellow (*r* = 0.41, *P* = 0.004) and negatively correlated with the module MEmidnightblue (*r* = -0.41, *P* = 0.004) (Fig 6F). Modules MEpink, MEturquoise, and MEyellow were also significantly correlated with ARW variation at *P* < 5.0E-09 (Fig 6F).

### Modules highly correlated with OY

**Fig 7:**
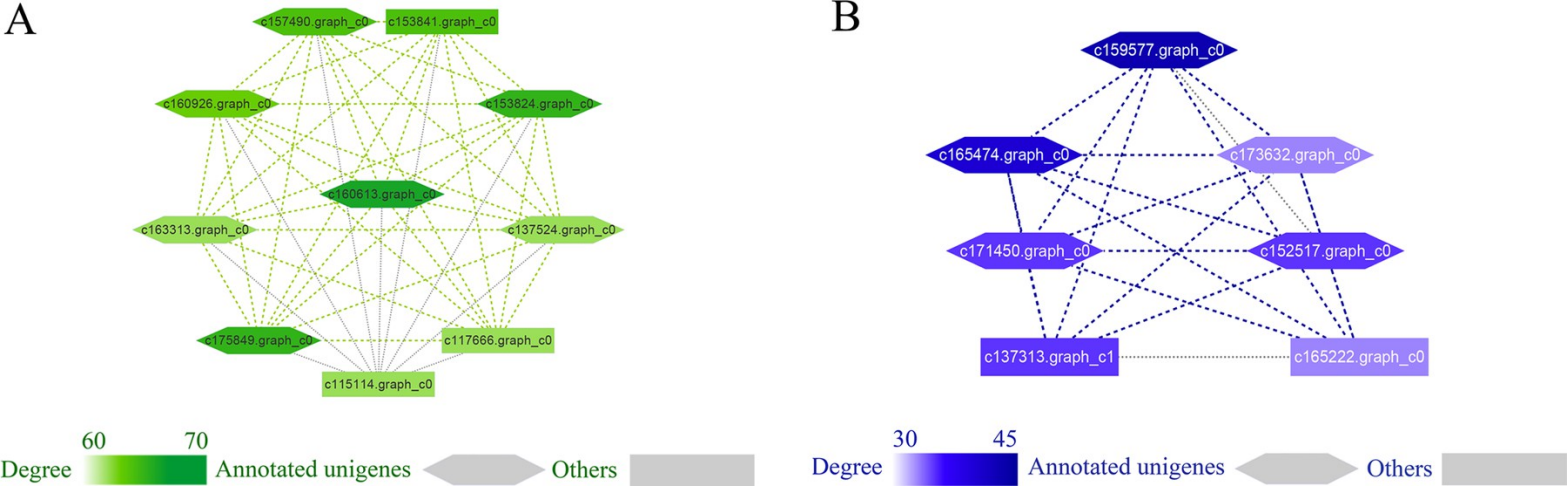
Hub gene visualization of the MEgreenyellow and MEmidnightblue modules associated with OY. **A** Ten hub genes from MEgreenyellow module were included in the Cytoscape-generated diagram. **B** Seven hub genes of the MEmidnightblue module were included in the Cytoscape-generated diagram. Darker color indicates greater degree value. The highlighted dotted line indicates a gene with edge weights >0.02.

The MEgreenyellow module showed a significantly positive correlation with OY (Fig 7A). GO enrichment analysis for MEgreenyellow module indicated a significant association with hydrolase activity, protein dephosphorylation, carbohydrate transport, glutamate decarboxylase activity, calcium ion transport, calmodulin, and others (S2 Fig). We classified the top 10% of genes with the most degrees in the module as hub genes (S8 Table). A total of 10 hub genes were identified in the MEgreenyellow module. The top-ranked hub gene c160613.graph_c0 encodes germin-like protein (GLP) of the cupin superfamily which is the most diverse plant protein in seed plants and is involved in plant responses to biotic and abiotic stresses. Conversely, eigengenes in the MEmidnightblue module were highly negatively correlated with OY (Fig 7B). GO terms in the module genes were associated with response to heat stress. HSP binding and very long-chain fatty acid metabolic process protein were also significantly enriched in the module (S3 Fig). Of the 7 hub genes identified in this module, four encoded HSP family members, and one encoded a TF gene which has a heat stress transcription factor function (S9 Table).

### Modules highly correlated with ARW

**Fig 8:**
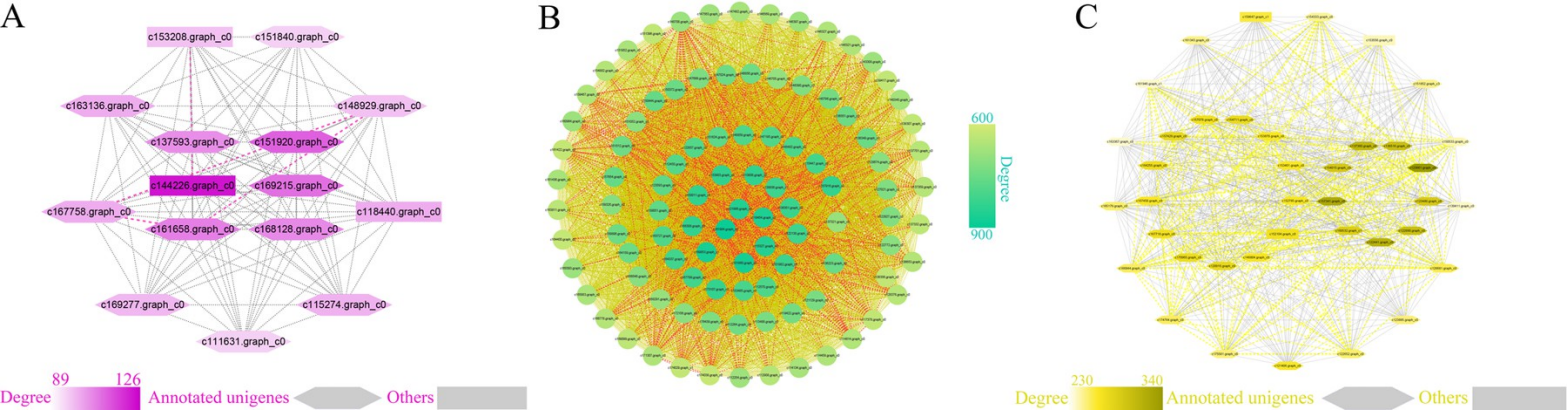
Hub gene visualization of MEpink, MEturquoise and MEyellow associated with ARW. **A** Fifteen hub genes of MEpink were included in the Cytoscape-generated diagram. **B** Ninety-nine hub genes of MEturquoise were included in the Cytoscape-generated diagram. **C** Thirty-nine hub genes of MEyellow were included in the Cytoscape-generated diagram. The darker the colour is, the greater the degree value. The highlighted dotted line indicates a gene with edge weights greater than 0.02.

The MEpink module had the most significantly association with ARW (Fig 8A). GO enrichment (S3 and S4 Files) of the first module identified genes associated with cell wall assembly, glutathione peroxidase activity and oxidoreductase activity. Most hub genes in the module were involved in developmental metabolic process and energy transport, such as acyl carrier protein pectate lyase, late embryogenesis abundant protein and glucose methanol choline oxidoreductase. The MEturquoise module was the largest module with 1060 eigengenes (Fig 8B). The GO categories mainly referred to biological processes and intermediates related to glucose and lipid metabolism that occur in different organelles. Functions of the 99 hub genes in this module were consistent with the results of GO enrichment analysis, such as protein tyrosine kinase, glycosyltransferase family, cytochrome b561, cytochrome c, inhibitor of apoptosis-promoting Bax1 and mitotic-spindle organizing protein 1. Notably, c136551.graph_c0 was annotated as a MYB86 TF that functions in root development and stomatal movement regulation in *Arabidopsis thaliana* [48]. The MEyellow module was also highly correlated with ARW (Fig 8C). GO analysis exhibited the enrichment of endoplasmic reticulum, hydrolase activity and cytokinesis. Interestingly, three hub genes were replication factors, including two replication factor-A proteins and one DNA replication licensing factor MCM7. Two hub genes were TF genes including bZIP and CBF/NF-Y.

### qRT-PCR verification

**Fig 9:**
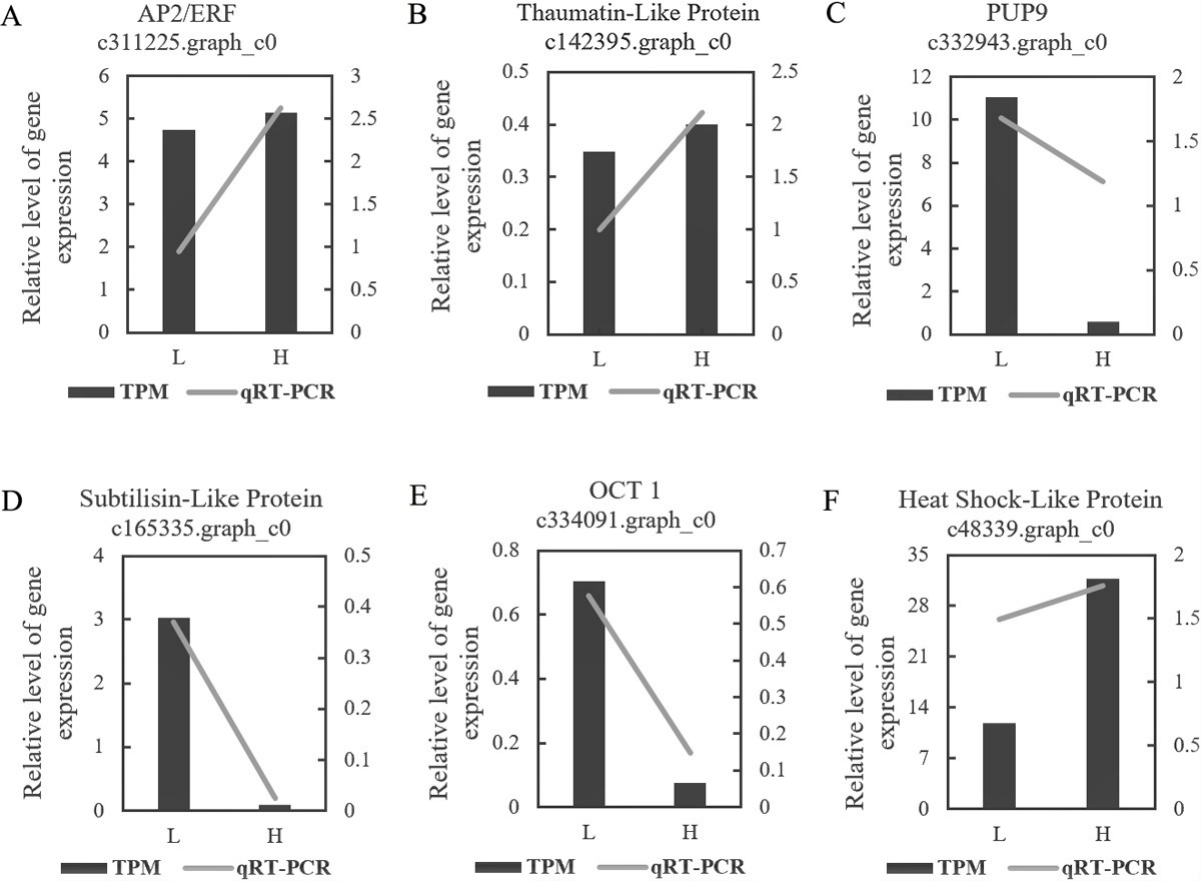
qRT-PCR validation of candidate genes associated with oleoresin yield and growth traits. The column shows the results of RNA-seq, and the connecting line shows the results of qRT-PCR.

To further determine transcript expression accuracy, six genes (c311225.graph_c0, c142395.graph_c0, c332943.graph_c0, c165335.graph_c0, c48339.graph_c0, c334091.graph_c0) were used for verification (S10 Table). All quantitative results corresponded well with the expression levels (Fig 9). A t-test confirmed that all the results were extremely significant, except the two genes (c311225.graph_c0, c142395.graph_c0) related to OY (S10 Data).

## Discussion

### Genetic variation is critical for successful tree breeding and association study

Genetic diversity matters for long-term tree breeding progress. Most conifer tree breeding programs only had 2–4 cycles of breeding selection [49]. Genetic structure and diversity of breeding population have not been greatly altered in forest trees with such slow cycle of selection [50, 51]. A slash pine breeding program aimed to improve timber and resin yields has been implemented for the past four decades with two cycles of selection. We observed that genetic diversity of the breeding population represented by the SNPs expected heterozygosity (*H*_*e*_ = 0.2565) was high and similar to the mean *H*_*e*_ of 0.228 for a widely distributed conifer species in natural population [52]. We also observed that the average inbreeding coefficient (*F*_*is*_) is low at 0.1373, suggesting that frequent inbreeding events did not occurred. This indicates that there are plenty genetic variation in the current breeding population for long-term and breeding and large genetic variation for association study. Even the genetic variation available in selected materials may be slightly less than that in natural population [53], the acculumated long-term and large amount of phenotypic data from tree breeding program are precious and ideal genetic data for dissecting the genetic basis of complex traits [17].

### Genetic structure of *slash pine* population resources in China and population size for association study

There were several procurements of slash pine seeds from USA for Chinese tree breeding program in the recent half century, however, population origin and structure were unknown for several import. Population structure will affect breeding selection efficiency and association studies [54]. Our analysis revealed that three main genetic groups for the current Chinese breeding population, there might be small subpopulations within group 1 and 2 populations. Such information were used in our association study and will assist our genetic evaluation and breeding value prediction for our next generation of breeding.

Population size is the most important factor in association study. Power calculation using a method developed for trees [55] indicated that under a significance level of 1 x 10^−7^ and 80% power (standard in GWAS), 50% genetic variation for polygenic traits having heritability >0.5, and controlled by up to about 9, 20, and 35 quantitative trait loci (QTL) will be captured by a population size of 250, 550 and 850, respectively, and individual QTLs having effect sizes of about 12%, 6.2% and 4% of total genetic variation will be most likely detected, corresponding to the three sizes of populations. We indeed capture 32 SNPs of large effect with QTL effect from 10.6% to 21.8% in this study. To increase detection for QTL with smaller effect, our population size should be increased to 500 or 1000 in order to capture QTLs of small effect (< 5% phenotypic variation).

### Association analysis based on RNA-seq shows strong potential

We found only a few associated QTLs by TWAS, which is very common in plant species. However, several significant SNPs have significant function annotation. Two SNPs (sc311225.graph_c0_seq1_529; sc142395.graph_c0_seq1_178) significantly associated with OY were annotated as PeAP2/ERF and PeTLPs, respectively [56]. In loblolly pine, both SNPs in the AP2/ERF domain and in its downstream gene ETHYLENE INSENSITIVE2 (EIN2) were associated with OY [41]. This may suggest that AP2/ERF is an important candidate gene that regulate the oleoresin yield. It is well known that AP2/ERF responds to the plant hormones abscisic acid (ABA) [57] to activate ABA-dependent and ABA-independent stress-responsive genes. AP2/ERFs are implicated in growth and developmental processes mediated by gibberellins [58], (CKs) [59], and brassinosteroids [60]. Another example is SNPs in TLPs. TLPs belong to the PR-5 family which were widely found in higher plants. Meng et al. [61] found that TLPs play an important role in plant antibacterial activity by influencing α-pinene content to reduce the damage to *P*. *massoniana* [61], *P*. *taeda* [62] and *P*. *sylvestris* [63] caused by pests.

Three SNPs loci associated with Ht are annotated as PeSLP, PeHSP and PeOCT1 respectively. SLP can catalyze the hydrolysis of related proteins during the first stage of cytoderm degradation and promote cell elongation [64]. The *ALE1* gene of the SLP family in *Arabidopsis* is required for cuticle formation in the protoderm [65]. SLP may also be involved in xylem development in a number of interesting ways, such as molecular triggers or downstream effectors of programmed cell death, or possibly by acting as processing enzymes of peptide hormones [66]. HSP was found having important function of promoting cell division and cell elongation in plant [67,68]. The organic cation/carnitine transporter 1 (OCT 1) disruption in an Arabidops knockout mutant affects both the expression of carnitine-related genes and the development induced by exogenous carnitine, showing that AtOCT1 disruption affects root development under certain conditions [69].

Three SNPs significantly associated with DBH may link with genes having great potential of regulating tree radial growth. The SNP sc336796.graph_c0_seq1_427 was annotated as expansin-like protein (ELP). Growing tissues of most plants have been shown to undergo acid-induced extension, and it is generally accepted that ELPs are the chief agents responsible for acid-induced extension [70]. Darley et al. [71] pointed out that ELPs play a variety of roles in vivo by modifying the cell wall matrix during growth and development. Interestingly, ELPs appear to increase polymer mobility in the cell wall, allowing the structure to slide apart during extension [72]. Surprisingly, both the quantitative and transcriptome results in this study revealed that individuals with larger DBH have lower PeELP gene expression. This may indicate that individuals with a smaller DBH have greater growth potential with higher PeELP expression. Two SNPs including sc332943.graph_c0_seq1_514 and sc332943. graph_c0_seq1_348 that were significantly associated with DBH were annotated as PePUP9 in this study. There is evidence that AtPUP2 may mediate the long-distance transport of adenine [44]. Qi et al. [72] stated that OsPUP7 in rice had a transport function similar to that of AtPUPs and indicated that rice also has a similar PUP transport system to transport CKs or their derivatives. These findings of cell division, elongation, stress and transportation related gene would be great interest to futher verification for functional studies and breeding purposes. The verified QTLs could be used to increase efficiency of genomic selection [73].

### Transcriptome expression profiles provide a complementary approach to association analysis

Transcriptome expression profiles quantify transcript abundance, which provides a complementary approach to GWAS. The detection of associations between gene expression and trait variation was particularly useful in studying complex traits of polyploid organisms [12]. In wheat, the application of associative transcriptomics identified associations between trait variation and both SNPs and GEMs [74]. Methyltransferase and tocopherol cyclase that were significantly (*P* < 0.01) related to OY play an important role in the biosynthesis of irregular terpenes [75], regulation of cell apoptosis [76,77], and plant defense mechanisms [78]. The annotation results of genes related to slash pine growth traits show that most genes and TFs with the same or similar functions shared among the three growth traits. These include cytochrome P450 [79], F-box domain [80], G-box binding factor [81], NAM [82] and the TFs of Trihelix [83], bHLH [84], MADS-box [85], GATA [86], and MYB [87]. Particularly, the Trihelix TF family is related to all three growth traits of slash pine and this family performs the functions of development of perianth organs, trichomes, stomata, the seed abscission layer, and the regulation of late embryogenesis [83,88]. Many members of the bHLH family that mediate axillary bud development, spike initiation [84], and iron homeostasis regulation [89], were also related to slash pine three growth traits.

### Co-expression network identified the hub genes controlling OY and ARW

Wang and colleagues [90] used a population-associative transcriptomic approach to identify genes related to spike complexity. Consistent with the GWAS results, we identified a hub gene significantly associated with OY in the MEgreenyellow module, which encodes a pathogenesis-related thaumatin superfamily protein. GLPs of the cupin superfamily were also found in this module and in the GEM-based correlation analysis for two growth traits of Ht and DBH. These are the most diverse plant proteins in seed plants and are involved in plant responses to biotic and abiotic stresses [91]. Cupin superfamily proteins are also involved in the regulation of seed germination and seedling development [92], which was repeated in our results of the GEM-based correlation analysis for the two growth traits of Ht and DBH. Notably, five of the seven hub genes in the MEmidnightblue module, which exhibited a negative correlation with OY, were annotated as HSP, and one was HSP TF. The reason for the negative correlation between HSP and OY may be that slash pine increased terpenoids secretion after being subjected to cold and heat stress, thereby reducing the synthesis of HSP. A study of in *Solanum lycopersicum* revealed that the emissions of mono- and sesquiterpenes gradually increased with the severity of cold or heat shock stress [93]. Consistent with the GEM-based results, we also found hub genes that were significantly related to ARW in the MEturquoise and MEyellow modules. These genes encode cyclin [94] and MYB [87], bZIP [95] and CBF/NF-Y [96] TFs, respectively, and perform important functions during plant development. Interestingly, HSP genes were significantly correlated with Ht in the TWAS analysis, but negatively correlated with OY in the MEmidnightblue module. The TLP gene was significantly associated with OY in the GWAS analysis and positively correlated with OY in the MEgreen module. We also used gene expression results of qRT-PCR to further verified function of these candidate genes and the reliability of RNA-seq analysis. The qRT-PCR findings confirmed that candidate genes expression levels were related to the development of secondary xylem tissues and oleoresin production.

## Supporting information

S1 Fig Cross validation error rate when K value of population structure is 3. The K value represents the number of preset population subgroups. The red dot in the figure represents the K value corresponding to the lowest error rate of cross validation. The population of slash pine is divided into three genetic groups.
(TIF)

S2 Fig GO enrichment analysis for eigengenes in MEgreenyellow module. The vertical axis represents the categories of GO enrichment analysis, and the horizontal axis represents the number of genes enriched in different categories. The colors from blue to red indicate increasing significance.
(TIF)

S3 Fig GO enrichment analysis for eigengenes in MEmidnightblue module. The vertical axis represents the categories of GO enrichment analysis, and the horizontal axis represents the number of genes enriched in different categories. The colors from blue to red indicate increasing significance.
(TIF)

S1 Table Phenotypic data of six traits of slash pine used for GWAS analysis. (XLSX)

S2 Table Function annotation of unigenes. (XLS)

S3 Table The quantity statistics of Unigenes with different length. (XLSX)

S4 Table Genetic diversity based on SNPs in the 240 slash pine individuals. (DOCX)

S5 Table The number of slash pine individuals in different sets in PCA results. (XLSX)

S6 Table The Admixture results were consistent with PCA analysis. (XLSX)

S7 Table Genetic diversity parameters at the group level inferred by ADMIXTURE. (DOCX)

S8 Table Hubgenes associated with OY in MEgreenyellow and MEmidnightblue module respectively. (XLSX)

S9 Table Functional annotation of 4 genes encoding HSP family members and 1 gene encoding HSF. (XLSX)

S10 Table Samples and target genes of slash pine for qRT-PCR validation. (XLSX)

S1 File GO enrichment analysis of genes identified by gem-based method. (RAR)

S2 File The significantly (*P* < 0.01) correlated unigenes based on transcriptome association analysis. (RAR)

S3 File GO enrichment analysis and functional annotation of ARW-associated hubgenes. (RAR)

S4 File GO enrichment analysis of hubgenes in modules associated with ARW. (DOCX)

S1 Data Source data for Fig 2. Transcriptome analysis of 240 selected slash pine trees.
(RAR)

S2 Data Source data for Fig 3. Genetic structure analysis of *P*. *elliottii* Engelm.
(RAR)

S3 Data Source data for Fig 4. SNP-based association analysis.
(RAR)

S4 Data Source data part 1 for Fig 5. GEM-based association analysis, as a Manhattan plot.
(RAR)

S5 Data Source data part 2 for Fig 5. GEM-based association analysis, as a Manhattan plot.
(RAR)

S6 Data Source data part 3 for Fig 5. GEM-based association analysis, as a Manhattan plot.
(RAR)

S7 Data Source data for Fig 6. Construction of gene co-expression networks with WGCNA.
(RAR)

S8 Data Source data for Fig 7. Hub gene visualization of the MEgreenyellow and MEmidnightblue modules associated with OY.
(RAR)

S9 Data Source data for Fig 8. Hub gene visualization of MEpink, MEturquoise and MEyellow associated with ARW.
(RAR)

S10 Data Source data for Fig 9. qRT-PCR validation of candidate genes associated with oleoresin yield and growth traits.
(RAR)
