# Supplementary figures and images for: A transcriptome-based association study of growth, wood quality, and oleoresin traits in a slash pine breeding population
Source: PLoS Genet. 2022 Feb 2;18(2):e1010017. doi: 10.1371/journal.pgen.1010017 (PMC8843129; doi:10.1371/journal.pgen.1010017)

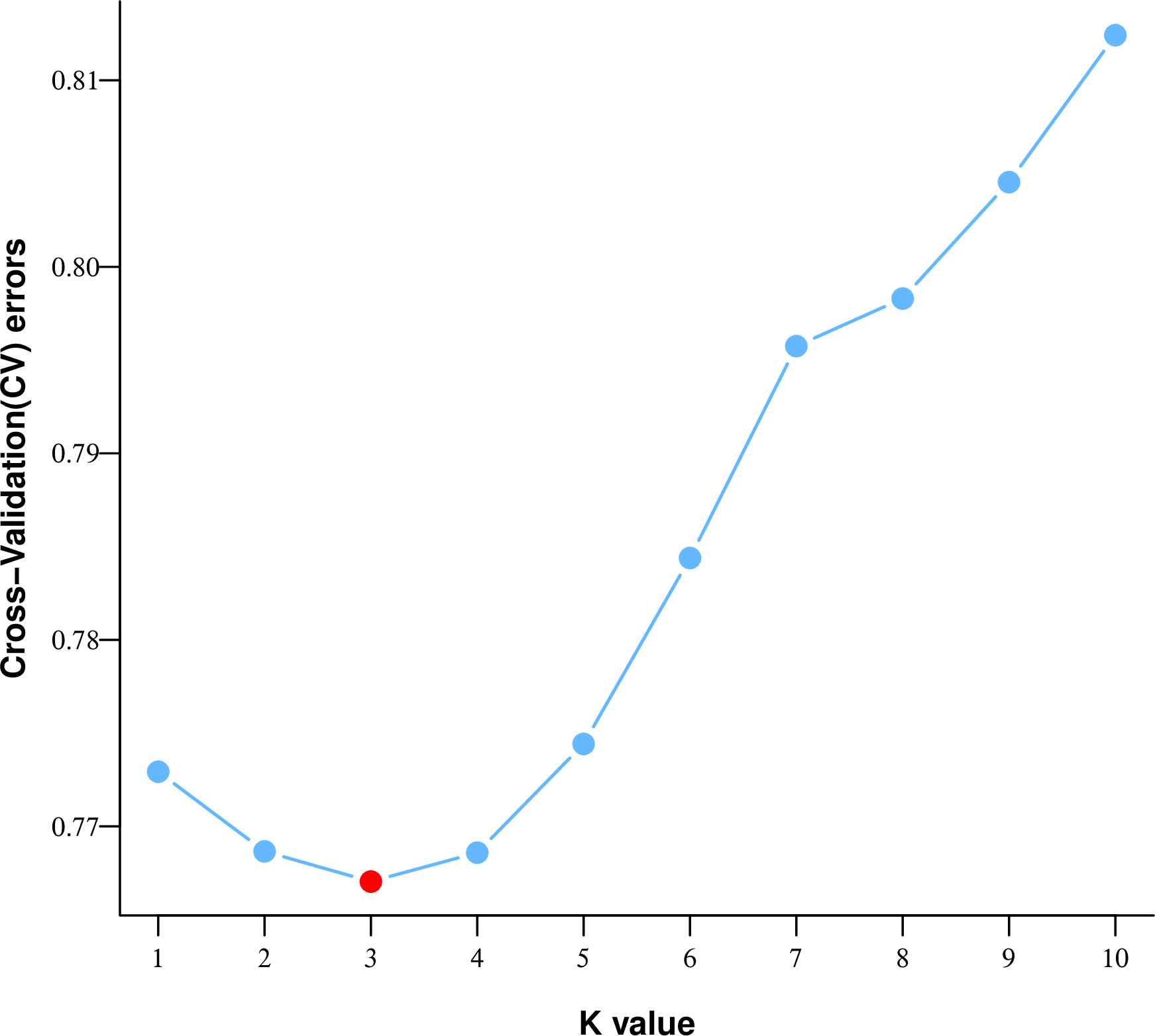

Supplement: S1 Fig — The K value represents the number of preset population subgroups. The red dot in the figure represents the K value corresponding to the lowest error rate of cross validation. The population of slash pine is divided into three genetic groups. (TIF) [file pgen.1010017.s001.tif]

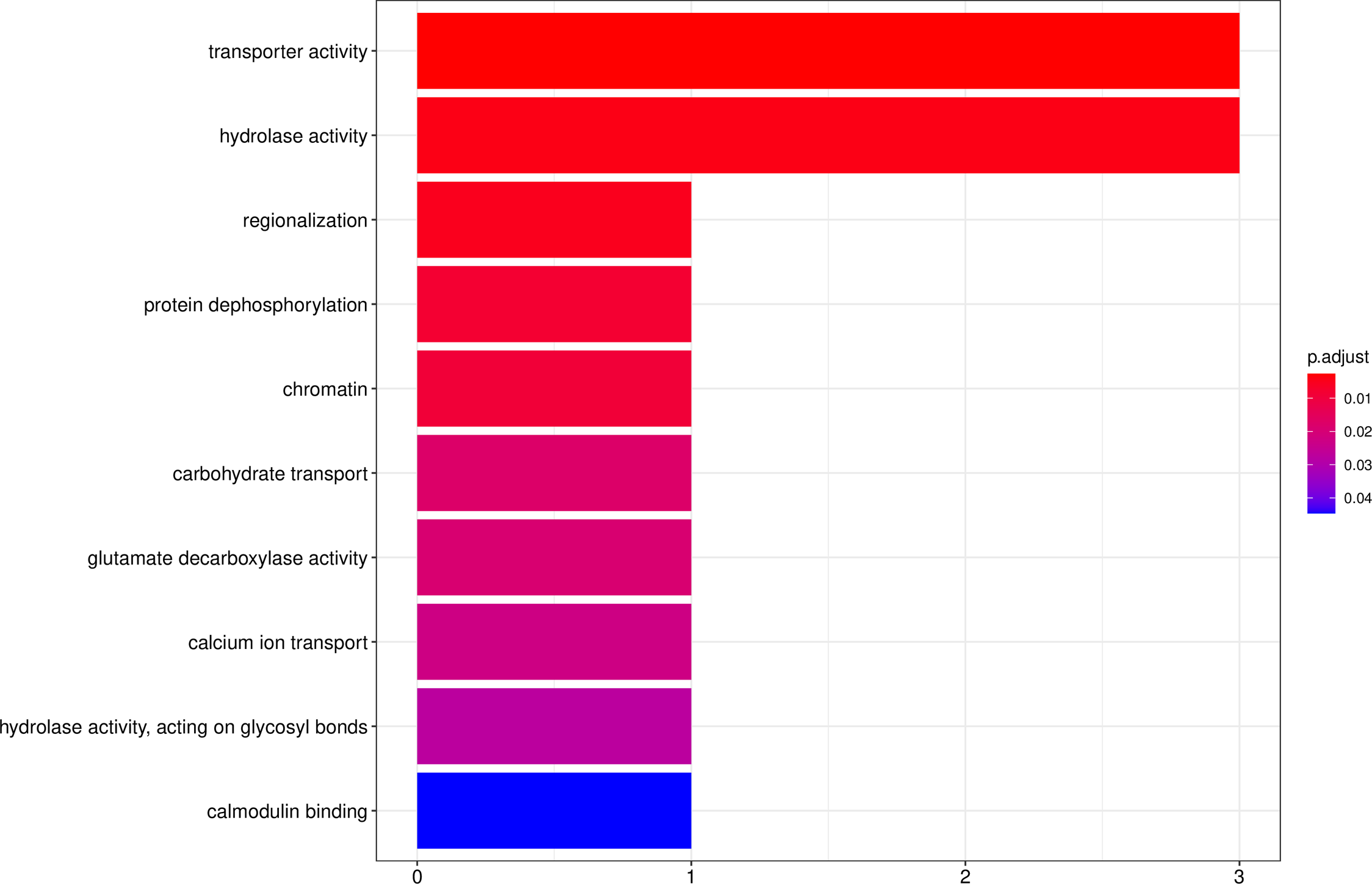

Supplement: S2 Fig — The vertical axis represents the categories of GO enrichment analysis, and the horizontal axis represents the number of genes enriched in different categories. The colors from blue to red indicate increasing significance. (TIF) [file pgen.1010017.s002.tif]

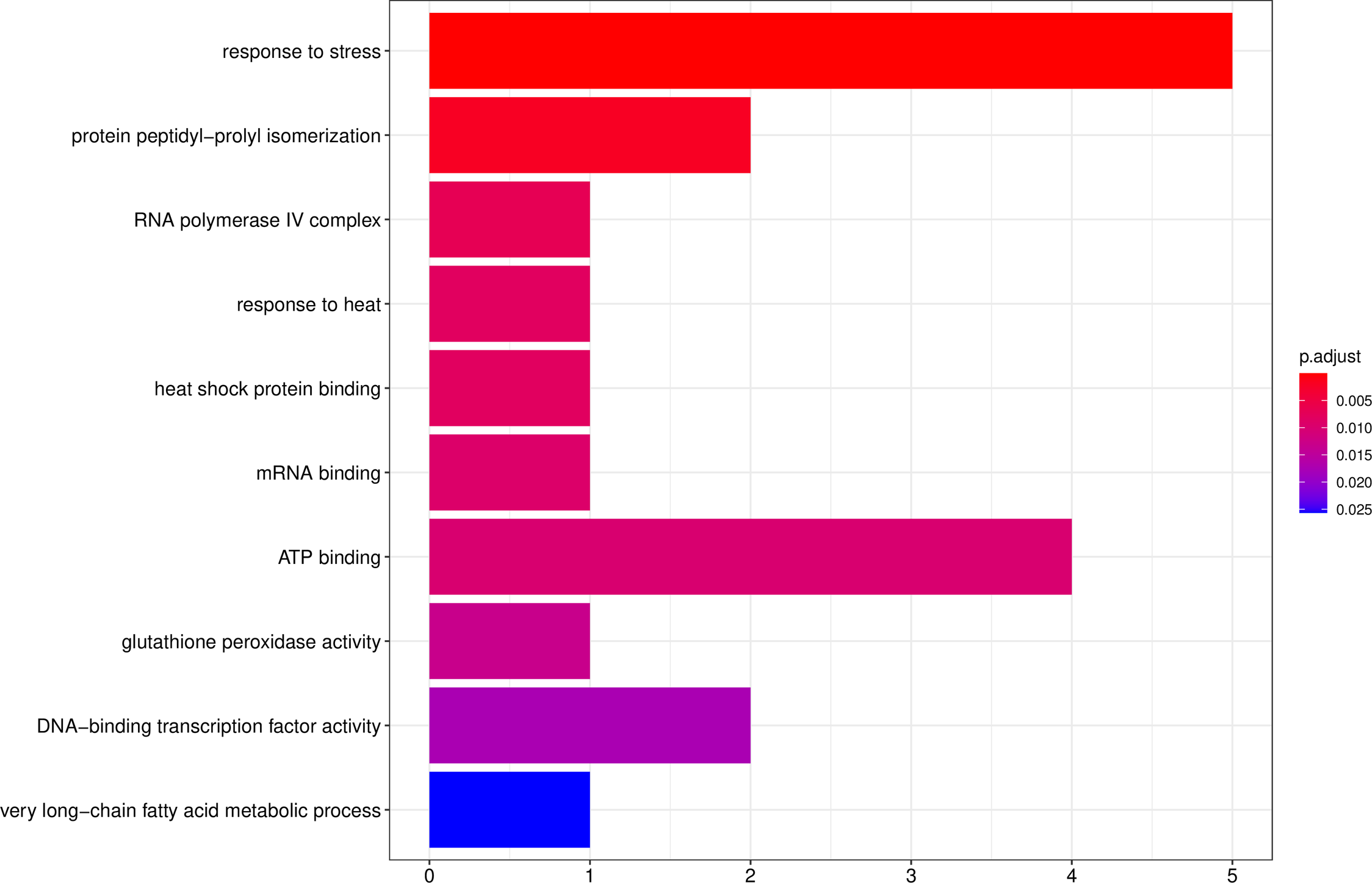

Supplement: S3 Fig — The vertical axis represents the categories of GO enrichment analysis, and the horizontal axis represents the number of genes enriched in different categories. The colors from blue to red indicate increasing significance. (TIF) [file pgen.1010017.s003.tif]
