# Supplementary material for: A transcriptome-based association study of growth, wood quality, and oleoresin traits in a slash pine breeding population
Source: PLoS Genet. 2022 Feb 2;18(2):e1010017. doi: 10.1371/journal.pgen.1010017 (PMC8843129; doi:10.1371/journal.pgen.1010017)
Supplement: S4 Table — (DOCX) [file pgen.1010017.s007.docx]

**S4 Table.** **Genetic diversity based on SNPs in the 240 slash pine individuals.**

| **Variation** | **Min** | **Max** | **Average** | **CV** |
| --- | --- | --- | --- | --- |
| H_o_ | 0.0815 | 0.3622 | 0.2246 | 0.1127 |
| H_e_ | 0.2556 | 0.2584 | 0.2565 | 0.0011 |
| N_e_ | 1.0888 | 1.5679 | 1.2911 | 0.0339 |
| F_is_ | 0.0108 | 0.3325 | 0.1373 | 0.7925 |

*H_o_* observed heterozygosity, *H_e_* expected heterozygosity, *N_e_* effective number of alleles, *F_is_* inbreeding coefficient, Min minimum values, Max maximum values, CV coefficient of variation.
