# Supplementary material for: A transcriptome-based association study of growth, wood quality, and oleoresin traits in a slash pine breeding population
Source: PLoS Genet. 2022 Feb 2;18(2):e1010017. doi: 10.1371/journal.pgen.1010017 (PMC8843129; doi:10.1371/journal.pgen.1010017)
Supplement: S7 Table — (DOCX) [file pgen.1010017.s010.docx]

**S6 Table. *Nei’s* (below diagonal) unbiased genetic distance and *F_st_* (above diagonal) calculated between the three groups inferred by ADMIXTURE.**

|  | Group 1 | Group 2 | Group 3 |
| --- | --- | --- | --- |
| Group 1 | - | 0.077 | 0.060 |
| Group 2 | 0.169 | - | 0.101 |
| Group 3 | 0.170 | 0.172 | - |
