# Supplementary material for: A transcriptome-based association study of growth, wood quality, and oleoresin traits in a slash pine breeding population
Source: PLoS Genet. 2022 Feb 2;18(2):e1010017. doi: 10.1371/journal.pgen.1010017 (PMC8843129; doi:10.1371/journal.pgen.1010017)
Supplement: S4 File — (DOCX) [file pgen.1010017.s017.docx]

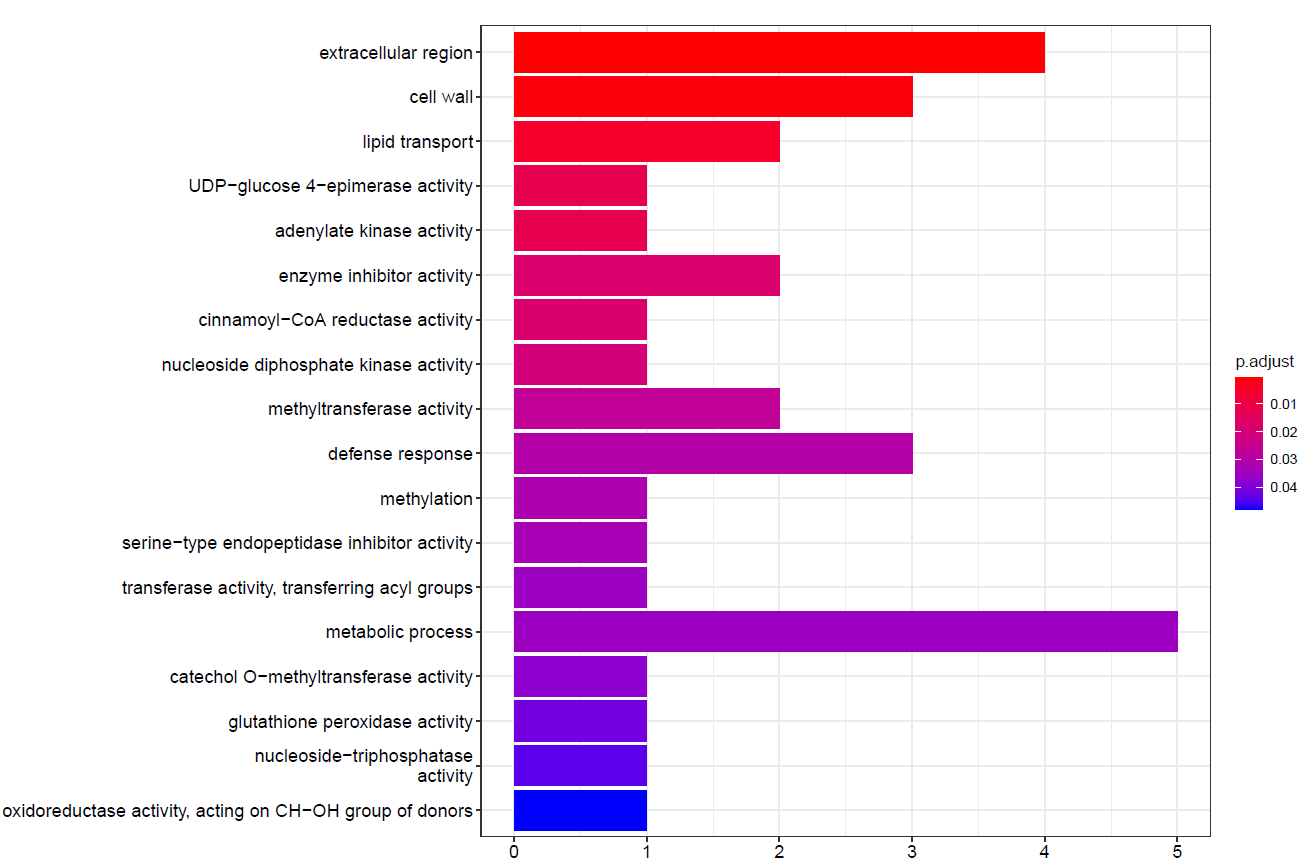


**S4_File 1. GO analysis of pink module genes**


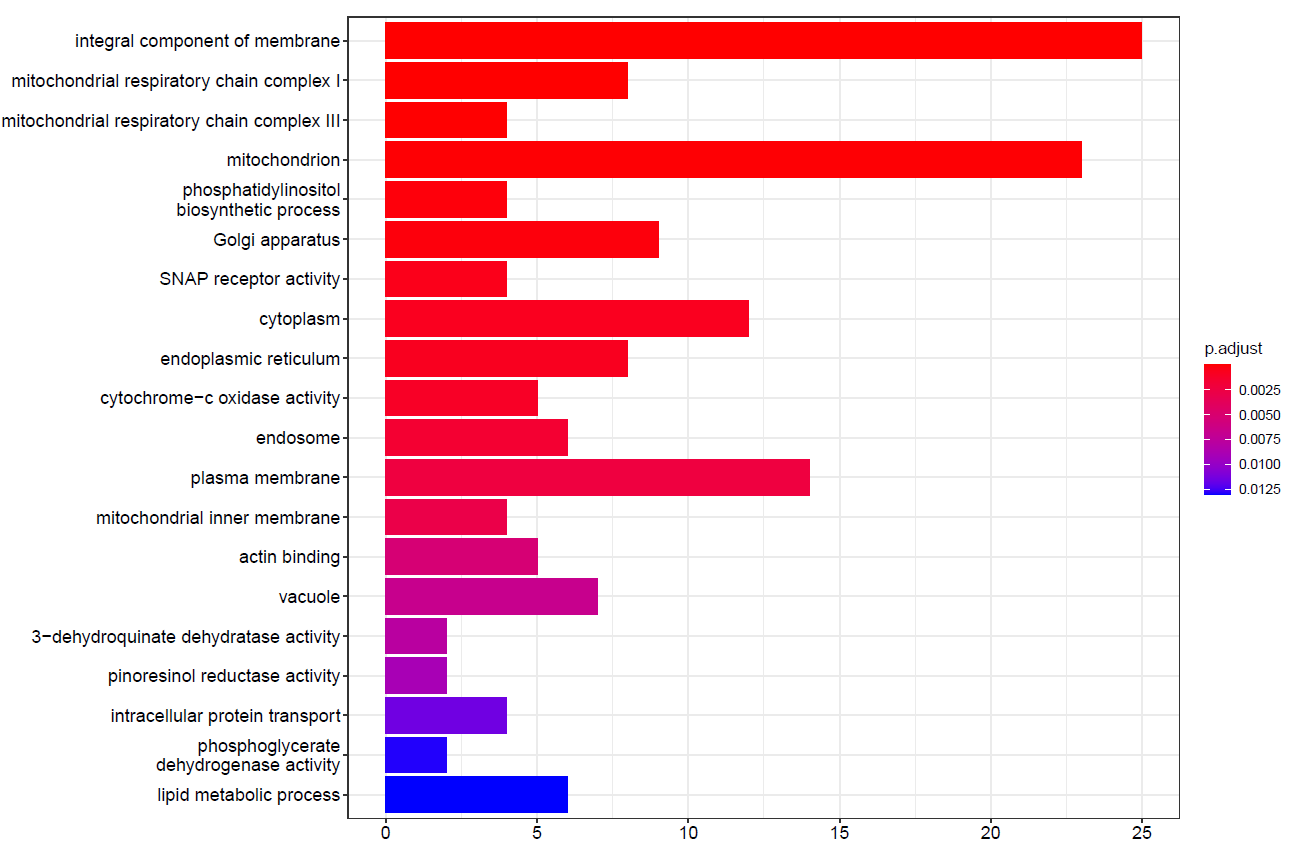


**S4_File 2. GO analysis of turquoise module genes**


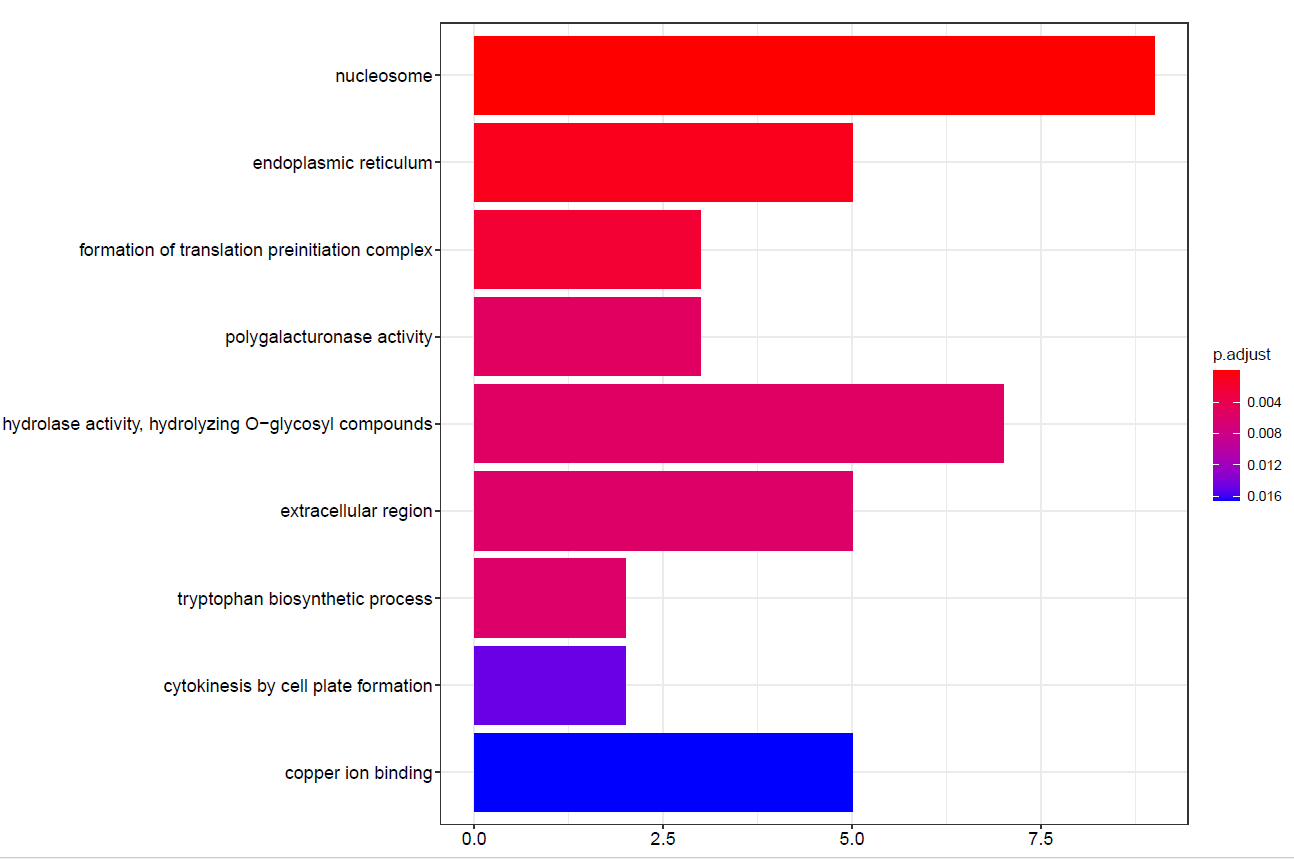


**S4_File 3. GO analysis of yellow module genes**
